# From percept to concept in the ventral temporal lobes: Graded hemispheric specialisation based on stimulus and task

**DOI:** 10.1016/j.cortex.2018.01.015

**Published:** 2018-04

**Authors:** Paul Hoffman, Matthew A. Lambon Ralph

**Affiliations:** a Centre for Cognitive Ageing and Cognitive Epidemiology (CCACE), Department of Psychology, University of Edinburgh, UK; b Neuroscience and Aphasia Research Unit (NARU), Division of Neuroscience & Experimental Psychology, School of Biological Sciences, University of Manchester, UK

**Keywords:** Anterior temporal lobe, Hub-and-spoke, Semantic knowledge, Lateralisation, Speech

## Abstract

The left and right ventral anterior temporal lobes (vATL) have been implicated as key regions for the representation of conceptual knowledge. However, the nature and degree of hemispheric specialisation in their function is unclear. To address this issue, we investigated hemispheric specialisation in the ventral temporal lobes using a distortion-corrected spin-echo fMRI protocol that enhanced signal in vATL. We employed an orthogonal manipulation of stimulus (written words *vs* pictured objects) and task (naming *vs* recognition). Words elicited left-lateralised vATL activation while objects elicited bilateral activation with no hemispheric bias. In contrast, posterior ventral temporal cortex exhibited a rightward bias for objects as well as a leftward bias for words. Naming tasks produced left-lateralised activation in vATL while activity for recognition was equal in left and right vATLs. These findings are incompatible with proposals that left and right ATLs are strongly modular in function, since these predict rightward as well as leftward biases. Instead, they support an alternative model in which (a) left and right ATL together form a bilateral, integrated system for the representation of concepts and (b) within this system, graded hemispheric specialisation emerges as a consequence of differential connectivity with other neural systems. On this view, greater left vATL activation for written word processing develops as a consequence of the inputs this region receives from left-lateralised visual word processing system in posterior temporal cortex. Greater left vATL activation during naming tasks is most likely due to connectivity with left-lateralised speech output systems in prefrontal and motor cortices.

## 1 Introduction

One key function of the ventral temporal cortices (VTC) is to act as a “ventral visual stream” that is critically involved in visual object recognition (Goodale and Milner, 1992, Ungerleider and Mishkin, 1982). In non-human primates, for example, posterior-to-anterior regions of the ventral occipitotemporal cortex are implicated in increasingly complex aspects of visual perception, at the apex of which are neurons in anterior temporal cortex that code for object categories independent of view or other low-level characteristics (Albright, 2012, Booth and Rolls, 1998, Kravitz et al., 2013). In humans, however, the role of the anterior portion of VTC (which we will refer to as the ventral anterior temporal lobe, or vATL) extends far beyond the visual domain. Convergent evidence indicates that the vATLs are involved in semantic processing of visual objects and faces, but also names, concrete and abstract words, auditory speech and non-verbal sounds (Mion et al., 2010, Nobre et al., 1994, Pobric et al., 2009, Rice, Hoffman et al., 2015, Shimotake et al., 2015, Spitsyna et al., 2006, Visser et al., 2012). These data have led us and others to propose a model of the functional anatomy of the temporal lobes, in which the vATLs act as an integrative “hub” for the development of transmodal conceptual representations (Binney et al., 2012, Guo et al., 2013, Lambon Ralph et al., 2017, Patterson et al., 2007, Rice, Hoffman et al., 2015). On this view, high-level visual perceptual (in posterior VTC), auditory perceptual (in superior temporal cortex) and social and emotional processing streams (in orbitofrontal and temporopolar cortex) converge on vATL, which generates supramodal representations of concepts that bind these information sources together.

In this study, we investigated hemispheric specialisation in the function of the vATLs and compared this directly with visual processing specialisations in posterior VTC. There is already clear evidence from neuropsychological, neurostimulation and neuroimaging studies that both left and right vATLs make important contributions to concept representation (Butler et al., 2009, Gainotti, 2012, Humphreys et al., 2015, Lambon Ralph et al., 2012, Mion et al., 2010, Shimotake et al., 2015). However, there is an ongoing debate over the degree to which the function of each vATL is specialised for particular sensory modalities, conceptual categories or tasks (Gainotti, 2011, Gainotti, 2012, Gainotti, 2014, Drane et al., 2013, Rice, Hoffman et al., 2015). In considering these possible specialisations, a useful starting point is to consider hemispheric specialisation in posterior VTC. Visual processing in posterior VTC is bilateral but exhibits hemispheric specialisations, most notably a left-hemisphere bias for word recognition and a right-hemisphere bias for face and object recognition (Cohen and Dehaene, 2004, Hasson et al., 2002, Puce et al., 1996, Thierry and Price, 2006). Importantly, these distinctions are graded rather than absolute. Patients with left posterior VTC lesions exhibit severe deficits in word recognition but are also impaired in face recognition. In patients with right posterior VTC damage, the situation is reversed: face recognition is most severely affected but word recognition deficits are also present (Behrmann and Plaut, 2014, Roberts et al., 2013).

There is some evidence and debate that this form of graded hemispheric specialisation might extend anteriorly into the ATL region, including vATL. To date, the principal source of data in this debate has been neuropsychological, with minimal information coming from fMRI studies of healthy participants (which is the core target of the present study). Patients with predominately left-hemisphere ATL damage have greater difficulty comprehending written words compared with pictures or faces, while the reverse is true of right ATL damage (Butler et al., 2009, Gainotti, 2007, Snowden et al., 2004). However, these dissociations are graded rather than absolute and both sets of patients have significant difficulty with both classes of stimuli. These findings have led some researchers to propose that left ATL is specialised for representation of verbal concepts and right ATL for non-verbal concepts (Gainotti, 2012, Gainotti, 2014, Snowden et al., 2012).

Another factor potentially driving specialisation is the retrieval of lexical labels based on semantic information. Patients with left ATL damage have greater difficulty naming pictures and faces than do patients with right ATL damage (Acres et al., 2009, Damasio et al., 2004, Drane et al., 2013, Lambon Ralph et al., 2001, Lambon Ralph et al., 2012), which may be a consequence of the greater connectivity between left ATL and left-lateralised speech output regions (Rice, Hoffman et al., 2015; Schapiro, McClelland, Welbourne, Rogers, & Lambon Ralph, 2013). A recent TMS study has also shown greater effects of left ATL disruption on picture naming tasks, relative to right ATL (Woollams, Lindley, Pobric, & Hoffman, 2017). In contrast, some studies suggest that damage to right ATL has a disproportionate effect on visual recognition tasks that do not require retrieval of a name (Damasio et al., 2004, Drane et al., 2013). These findings have led some researchers to claim that left ATL is specialised for lexical retrieval (i.e., naming) from visual information while the right ATL plays a greater role in visual discrimination tasks (Drane et al., 2013). It is not clear whether these laterality effects occur upstream in posterior VTC.

Although they have been influential in identifying potential sources of hemispheric specialisation, the patient studies described thus far are limited in terms of anatomical specificity. Most studies involve either patients with semantic dementia or patients with temporal lobe epilepsy undergoing ATL resection. The lesions in these conditions invariably encroach on the temporal pole and on the lateral and superior aspects of the ATL, as well as the vATL region that is the focus of the present study (Galton et al., 2001). This is important because there is emerging evidence for functional specialisation across the broader ATL region, with the superior temporal lobe in particular showing a markedly different pattern of functional and structural connectivity to the rest of the ATL, as well as greater specialisation for auditory-verbal semantic processing (Jackson et al., 2016, Murphy et al., 2017, Pascual et al., 2013, Visser and Lambon Ralph, 2011). In addition, it is difficult to rule out potential reorganisation of function in individuals with chronic neurological disease. Functional neuroimaging studies in healthy individuals provide a complementary source of evidence, with the potential for a greater level of anatomical precision. We recently conducted a meta-analysis of 97 functional neuroimaging studies that reported ATL activations during semantic processing (Rice, Lambon Ralph, & Hoffman, 2015). While ATL activation was most commonly bilateral, we found (a) that left ATL activation was more likely than right for studies that used written words as stimuli and (b) that left ATL activation was more likely than right for naming tasks. However, no complementary right-hemisphere biases were observed for studies presenting pictures or using tasks other than naming. Thus, the extant neuroimaging literature is only partially consistent with the neuropsychological literature.

In addition, the meta-analytic data are subject to a number of caveats that stem from the nature of the studies we were able to include. Specifically:

1. In common with the neuropsychological literature, many of the pictorial neuroimaging studies presented faces rather than objects as stimuli. This is important because conceptual knowledge for people/faces might be represented differently to other object categories, either because of their status as unique entities or because they have strong social and emotional connotations. For example, face processing selectively activates polar regions of the temporal lobes that are strongly connected to limbic areas involved in emotion processing (Mehta et al., 2016, Simmons et al., 2010). In addition, while a clear right-hemisphere ATL bias has often been observed for processing of meaningful faces, it is less clear to what degree other object categories show a similar bias.
2. In the meta-analysis, we conducted statistical tests comparing the likelihood of obtaining left versus right ATL activations for each type of study. Meta-analyses of this kind take into account the presence or absence of an activation peak in each ATL but are not sensitive to cases where significant activation is present in *both* ATLs but with differing effect sizes. A more sensitive test requires direct within-subjects statistical comparison of left and right-hemisphere effect sizes in a single study. Unfortunately, such between-region contrasts of activation are not routinely performed (Peelle, 2012).
3. Finally, and most importantly, the meta-analysis found little evidence of activation in the vATL and thus had limited power to detect hemispheric specialisation in this key region. This lack of activation stems from various technical issues (Visser, Embleton, Jefferies, Parker, & Lambon Ralph, 2010), most notably poor fMRI signal in the vATL region due to the proximity of air-filled sinuses (Ojemann et al., 1997) as well as the use of low-level functional baselines (i.e., rest, see Humphreys et al., 2015) and limited field-of-view. Other techniques that do not suffer from this limitation (e.g., PET, MEG, eCog) report vATL activity much more reliably during all forms of semantic processing (Devlin et al., 2000, Marinkovic et al., 2003, Shimotake et al., 2015), as do recent fMRI studies that use acquisition methods that improve signal in this region (Halai et al., 2015, Hoffman, Binney et al., 2015, Humphreys et al., 2015, Jackson et al., 2015). Since relatively few fMRI studies have used these techniques, however, neuroimaging data on laterality effects in the ventral portion of the ATLs is scarce. Indeed, it is possible that hemispheric specialisation within the broader ATL region is driven by a left-hemisphere bias for words in its more superior lateral aspects (e.g., Vandenberghe et al., 1996, Visser and Lambon Ralph, 2011) and does not extend into vATL.

In the present study, we investigated hemispheric specialisation across VTC, including vATL, using an orthogonal manipulation of stimulus type (written word *vs* picture) and task (naming *vs* recognition). We improved sensitivity to vATL activity by using a distortion-corrected spin-echo fMRI protocol (Embleton, Haroon, Morris, Lambon Ralph, & Parker, 2010). We took a region-of-interest approach in which we divided each VTC into a series of slices extending from temporooccipital cortex forward towards the temporal pole. This allowed us to (a) directly compare left and right-hemisphere responses in each portion of VTC, including vATL, and (b) to assess how these laterality effects changed along the posterior–anterior axis of VTC.

## 2 Method

### 2.1 Participants

Twenty-seven healthy, right-handed participants took part (11 male, mean age = 25, range = 20–39). Data from one participant was discarded due to image artefacts. All participants were native English speakers with no history of neurological or psychiatric disorders and normal or corrected-to-normal vision. The study was approved by the local ethics board.

### 2.2 Design and experimental tasks

**Fig. 1:**
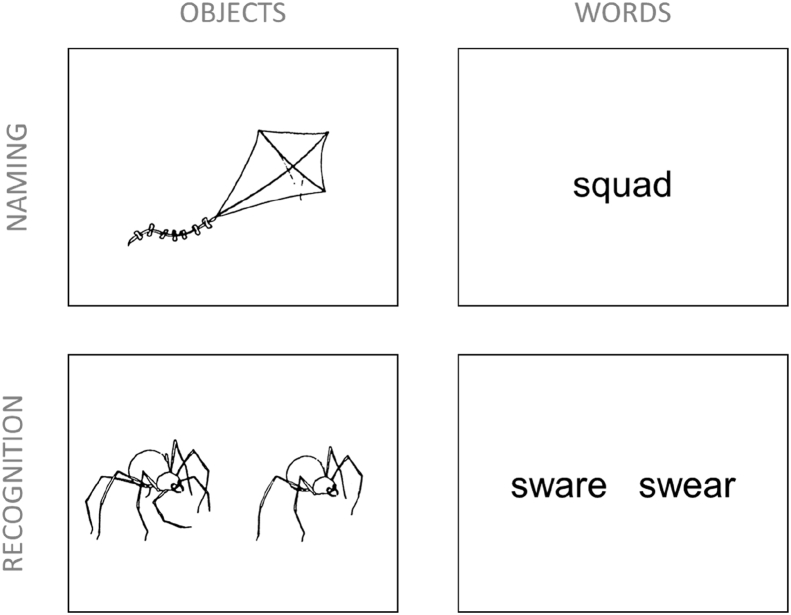
Tasks used in the scanner.

Participants completed four semantic tasks: object naming, word naming (reading aloud), word recognition and object recognition (see Fig. 1). This represented a 2 × 2 factorial manipulation of stimulus modality and task. In the object naming task, participants were presented with 144 line drawings of animals and manmade objects, taken mainly from the Snodgrass and Vanderwart (1980) set. They were asked to name each picture as quickly and accurately as possible. In the word naming task, participants were presented with 180 monosyllabic words from the Cambridge surface list (Patterson & Hodges, 1992) and asked to read them aloud as quickly as possible. Data from this task were the subject of a previous report (Hoffman, Lambon Ralph, & Woollams, 2015). The stimulus set contained a high proportion of words with irregular spelling-to-sound correspondences, which are believed to rely more heavily on semantic processing during reading (Woollams, Lambon Ralph, Plaut, & Patterson, 2007).

The word and object recognition tasks each employed 108 stimulus pairs from Hauk et al., 2007, Hauk et al., 2006. On each word recognition trial, participants were presented with a pair of letter strings and asked which represented a correctly-spelled English word. On each object recognition trials, participants were presented with two Snodgrass and Vanderwart images: a real object and a modified version that had an implausible feature. They were asked to indicate the real object. These recognition tasks were used because previous studies have shown that decisions presented in this way require semantic processing of the stimuli (Patterson et al., 2006, Rogers et al., 2004).

All stimuli were presented using Eprime software, in black on a white background. Word stimuli were presented in Arial font. Responses in the recognition tasks were made by button press, while a noise-cancelling MRI microphone (Optoacoustics) was used to record verbal responses. Recordings were manually coded offline for accuracy and reaction time. To minimise head movement, participants were asked to speak without moving their jaw (i.e., with teeth together).

Each task was performed in a separate scanning run with the order of tasks counterbalanced across participants. In each run, trials were presented in blocks of 13.5 s, with a rest block of 13.5 s following every fifth task block. For the object naming task, each block consisted of four stimuli (2875 ms per stimulus with inter-trial interval of 500 ms). For the word naming task, each block consisted of five stimuli (2200 ms per stimulus with inter-trial interval of 500 ms). For the word and object recognition tasks, each block consisted of three stimuli (4000 ms per stimulus with inter-trial interval of 500 ms). We used different trial durations for each task because pilot testing indicated that reaction times varied considerably across tasks (see Table 1 for confirmation of this). In order to approximately equate total time-on-task per block, we therefore presented the tasks with shorter RTs at a faster rate. It is important to note also that the four tasks were not designed to be matched in terms of visual complexity and that the overall activation elicited by each task might differ for this reason, particularly in posterior ventral temporal regions associated with visual processing. However, the principal comparisons of interest in this study were between left and right hemisphere activation to the same stimuli.

**Table 1:** Mean (standard deviation) behavioural performance for each task.

Task	% Accuracy	Reaction time, ms
Word naming	97 (1.9)	728 (94)
Object naming	94 (4.0)	1079 (112)
Word recognition	94 (4.3)	1334 (220)
Object recognition	96 (2.7)	1495 (201)

Each run also contained blocks of a non-semantic task, which were not of interest for the current analysis. The non-semantic tasks were as follows (see Supplementary Fig. 1 for examples). Object naming: view scrambled images and say “ok”. Word naming: view Greek character strings and say “ok”. Lexical decision: View two character strings and select the one containing only Roman letters. Object decision: View two boxes of characters and select the one containing only Roman letters.

### 2.3 Image acquisition and processing

Images were acquired on a 3T Philips Achieva scanner using an 8 element SENSE head coil with a sense factor of 2.5. The spin-echo EPI sequence included 31 slices covering the whole brain with echo time (TE) = 70 ms, time to repetition (TR) = 3200 ms, flip angle = 90°, 96 × 96 matrix, reconstructed in-plane resolution 2.5 × 2.5 mm, slice thickness 4.0 mm 896 images were acquired in total, in four runs of approximately 12 min. Following the standard method for distortion-corrected spin-echo fMRI (Embleton, Haroon, Morris, Lambon Ralph, & Parker, 2010), the images were acquired with a single direction k space traversal and a left-right phase encoding direction. In addition, a brief “pre-scan” was acquired, consisting of 10 volumes of dual direction k space traversal SE EPI scans. This gave 10 pairs of images matching the functional time series but with distortions in both phase encoding directions. These scans were used in the distortion correction procedure. In addition, a high resolution T1-weighted 3D turbo field echo inversion recovery image was acquired (TR = 8400 ms, TE = 3.9 ms, flip angle 8°, 256 × 205 matrix reconstructed to 256 × 256, reconstructed resolution .938 × .938 mm, and slice thickness of 0.9 mm, SENSE factor = 2.5) with 160 slices covering the whole brain.

The spatial remapping correction was computed using the method reported by Embleton et al. (2010). In the first step, each image from the main functional time-series was registered to the mean of the pre-scan images using a 6-parameter rigid-body transformation in SPM12. Subsequently, a spatial transformation matrix was calculated from the pre-scan images, consisting of the spatial re-mapping necessary to correct the distortion. This transformation was then applied to each of the 896 co-registered functional images.

Further image processing was performed using the FIACH toolbox, which is designed to reduce effects of head motion in studies that employ overt speech production (Tierney et al., 2016). The toolbox removes signal spikes in the time-series of individual voxels and uses principal components analysis to identify noise components in each participant's data across voxels. The first six noise components were included as covariates of no interest in first-level analyses. The motion and distortion-corrected images for each participant were co-registered to their T1 structural scan. Spatial normalisation of the T1 scans into MNI space was computed using DARTEL (Ashburner, 2007) and the resulting transformation applied to the functional images, which were resampled to 3 × 3 × 3 mm voxel size and smoothed with an 8 mm FWHM Gaussian kernel. At this point, temporal signal-to-noise (TSNR) maps were generated for each participant by dividing the mean signal in each voxel by its standard deviation (Murphy, Bodurka, & Bandettini, 2007). TSNR exceeded 100 in all parts of VTC (see Supplementary Fig. 2).

Following pre-processing, data were treated with a high-pass filter with a cut-off of 128 s and analysed using a general linear model with a block design. Each run included one semantic task and one non-semantic task. One regressor was used to model the semantic task and one for the non-semantic task (data from the non-semantic tasks were not analysed further). Blocks were modelled with a boxcar function convolved with the canonical hemodynamic response function. Motion and noise parameters were entered into the model as covariates of no interest.

### 2.4 Region of interest analyses

**Fig. 2:**
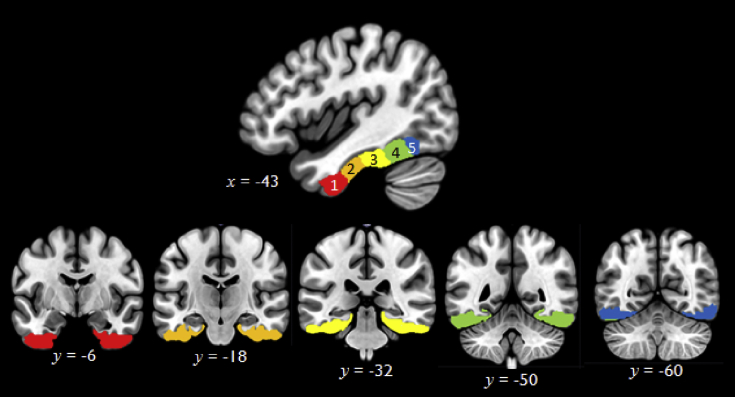
VTC regions of interest.

To analyse responses in left and right VTC, we created ten regions of interest based on the LPBA40 probabilistic brain atlas (Shattuck et al., 2008). We first constructed left and right VTC masks that included all voxels with a greater than 50% probability of falling within the inferior temporal, fusiform or parahippocampal gyri. We then divided each VTC into five ROIs by cutting them along the anterior–posterior axis, perpendicular to the long axis of the temporal lobe (see Fig. 2). The cutting points were selected so as to divide the VTC into five sections of roughly equal length; they were not constrained by any anatomical landmarks. The resulting ROIs, which we refer to slices, varied in volume (mean = 6487 mm^3^; range = 1904–8376 mm^3^). They were also not entirely symmetrical as they were determined by the LPBA40 probabilistic brain maps, which vary slightly across hemispheres.

Marsbar (Brett, Anton, Valabregue, & Poline, 2002) was used to extract median percent signal change in each ROI for each task and these data were then subjected to within-subjects ANOVA. The analysis was potentially complex because of the orthogonal manipulation of stimulus and task. To identify the best way to partition the data, we initially performed a 2 (hemisphere) × 2 (stimulus) × 2 (task) × 5 (slice) ANOVA. There was no stimulus × task interaction [*F*(1,25) = .005, *p* = .95] nor any interactions of stimulus × task with the other factors. We therefore divided the analysis into two parts, first investigating effects of stimulus (collapsed over task) and then investigating effects of task (collapsed over stimulus). In each case we used 2 (hemisphere) × 2 (stimulus or task) × 5 (slice) ANOVA to test for laterality effects over the entire VTC and for their interaction with slice location. We also used paired-samples *t*-tests to test for laterality effects in each individual slice for each stimulus/task. As this involved a large number of statistical tests, *p*-values were corrected for multiple comparisons using the false discovery rate approach (Benjamini & Hochberg, 1995). Finally, VTC slice #2 was of particular interest because it is the site of peak activations for multimodal semantic processing (e.g., Visser & Lambon Ralph, 2011) and thus is most informative about the function of vATL. To investigate effects in this slice in more detail, 2 (hemisphere) × 2 (stimulus or task) ANOVAs were performed.

## 3 Results

### 3.1 Behavioural performance

Each task was performed at over 90% accuracy (see Table 1). A 2 × 2 ANOVA on the accuracy data revealed no main effects of task or stimulus [*F*(1,25) < 2.7, *p* > .1] though there was a significant interaction [*F*(1,25) = 15.5, *p* = .001]. This reflects the fact that participants were significantly more accurate when naming words relative to objects [*t*(25) = 5.0, *p* < .001], whereas there was no effect of stimulus modality for the recognition tasks. An ANOVA performed on RT data indicated that participants were faster to respond on the naming tasks [*F*(1,25) = 358, *p* < .001] and faster to respond to words versus objects [*F*(1,25) = 75, *p* < .001]. There was also a significant interaction [*F*(1,25) = 21.5, *p* < .001], as the difference between word and object processing was larger in the naming tasks.

### 3.2 Effects of stimulus on left and right VTC activation

**Fig. 3:**
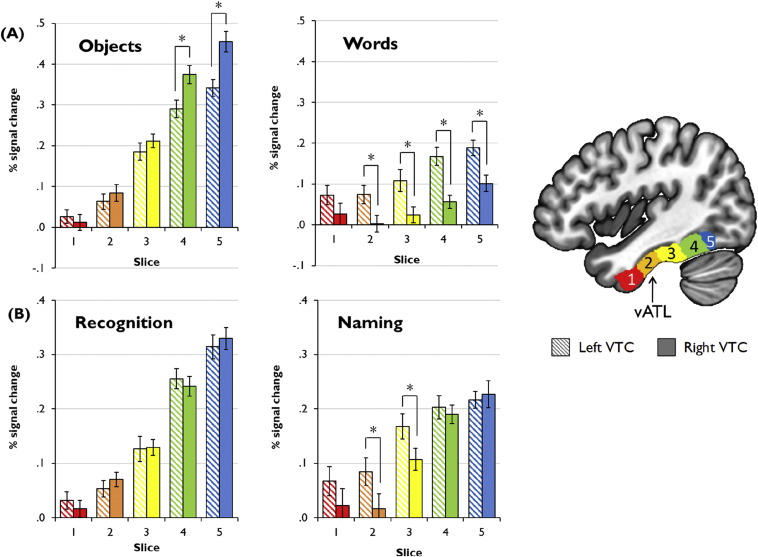
Activations for words, objects, naming and recognition in left and right VTC. * indicates significant difference between left and right VTC (FDR-corrected *p* < .05).

**Fig. 4:**
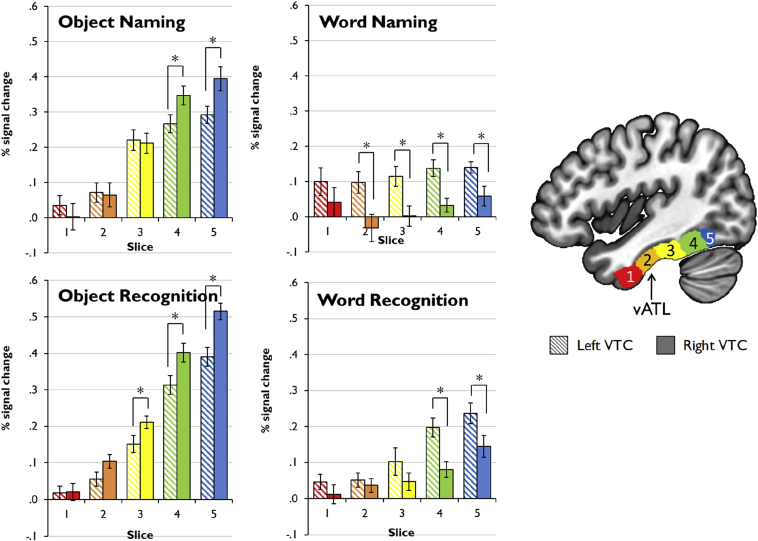
Activations in left and right VTC, broken down by individual task. * indicates significant difference between left and right VTC (FDR-corrected *p* < .05).

Fig. 3A shows percent signal change in response to words and objects in each slice of left and right VTC. Since our analyses collapsed over task, the plots in Fig. 3A are averaged over recognition and naming tasks. However, for completeness, in Fig. 4 we also provide a breakdown of activation for each individual task. As a general summary, we found that words evoked left-lateralised activation across the length of VTC, while activation for objects was right-lateralised in posterior VTC with no laterality effects in vATL. These effects were confirmed by a 2 (hemisphere) × 2 (modality) × 5 (slice) ANOVA. Although there was no main effect of hemisphere [*F*(1,25) = 3.47, *p* = .074], a main effect of slice was found [*F*(4,100) = 82.2, *p* < .001] reflecting larger signal changes in posterior VTC (though significant activation was also found in bilateral vATL). There was also a main effect of stimulus [*F*(1,25) = 60.9, *p* < .001]. Objects elicited stronger activation than words, likely due to their greater visual complexity. This effect was limited to posterior VTC, as evidenced by a significant stimulus × slice interaction [*F*(4,100) = 53.6, *p* < .001]. Importantly, there was an interaction of hemisphere with stimulus [*F*(1,25) = 47.1, *p* < .001], since word activation was left-lateralised while object activation tended to be right-lateralised. Finally, there was a three-way interaction [*F*(4,100) = 6.61, *p* < .001]. This reflects the fact that the left-hemisphere bias for words was maintained along the length of VTC while the right-hemisphere bias for objects was only present in posterior VTC.

A more detailed analysis of slice 2 (vATL) revealed a hemisphere × stimulus interaction in this location [*F*(1,25) = 9.60, *p* = .005]. Activation for words was greater in the left vATL relative to the right [*t*(25) = 2.75, *p* = .03] but there was no corresponding right-hemisphere bias for objects [*t*(25) = 1.15, *p* = .44].

### 3.3 Effects of task on left and right VTC activation

Fig. 3 shows percent signal change in response to naming and recognition tasks (collapsed over stimulus type) in each slice of left and right VTC. As a general summary, we found no laterality effects for the recognition tasks; however, a left-hemisphere bias was present for naming in mid-to-anterior VTC. These effects were confirmed by a 2 (hemisphere) × 2 (modality) × 5 (slice) ANOVA. There was no main effect of hemisphere [*F*(1,25) = 3.47, *p* = .074] but there was again a main effect of slice [*F*(4,100) = 82.2, *p* < .001]. There was no effect of task [*F*(1,25) = 3.59, *p* = .07] but there was a task × slice interaction [*F*(4,100) = 7.98, *p* < .001]. Recognition tasks produced larger signal changes than naming tasks in posterior parts of VTC. This effect is likely to be a consequence of differences in task design: two stimuli were presented on each trial for recognition tasks but only one was presented for naming. Differences in posterior VTC may therefore reflect greater visual processing demands for the recognition tasks. Importantly, there was an interaction of hemisphere with task [*F*(1,25) = 4.82, *p* = .038], since naming activation was biased towards the left hemisphere overall. The three-way interaction was not significant [*F*(4,100) = 1.83, *p* = .13].

A more detailed analysis of slice 2 (vATL) revealed a hemisphere × task interaction in this location [*F*(1,25) = 7.32, *p* = .012]. Activation for naming tasks was greater in the left vATL relative to the right [*t*(25) = 2.52, *p* = .046] but there was no corresponding right-hemisphere bias for recognition tasks [*t*(25) = .94, *p* = .55].

## 4 Discussion

We investigated hemispheric specialisation for visual-semantic processing in VTC, using a distortion-corrected fMRI protocol to maximise signal in the anterior portion of this region (i.e., the vATLs). vATL is known to play a critical role in the representation of conceptual knowledge but debate continues over the roles of left and right vATLs in the representation of verbal and non-verbal information and in naming versus recognition tasks (Drane et al., 2013, Gainotti, 2012, Gainotti, 2014, Rice, Hoffman et al., 2015). In posterior VTC, we found the well-established pattern of a left-hemisphere bias for written word processing and a right-hemisphere bias for visual object processing. These biases were only partially reproduced in the vATL: written words produced left-lateralised activation while pictured objects elicited equal activation in left and right vATLs. Similarly, partial graded specialisation was observed when naming and recognition tasks were contrasted. Naming elicited a left-lateralised response in vATL but no laterality effects were found during recognition tasks. These task-related effects were not observed in posterior VTC. Taken together, these findings are incompatible with a strong modular view of left and right vATL function. Instead, they are best accommodated by a model in which the bilateral vATLs function as an integrated system and graded specialisation emerges as a function of asymmetries in other neural systems to which they are connected (Lambon Ralph et al., 2017, Rice, Hoffman et al., 2015).

Our findings do not support strong modular distinctions between left and right ATLs, which necessarily require leftward *and* rightward functional biases. While we observed a left-hemisphere bias in the vATL response to words and naming tasks, there was no corresponding right-hemisphere lateralisation for either object processing or for recognition tasks. Instead, our findings support a model of ATL function in which the two ATLs form an integrated and partially redundant system for representing conceptual knowledge. This view is supported by a number of lines of evidence. First, while bilateral ATL damage has a severe effect on semantic processing, similar amounts of unilateral damage produce only mild semantic impairments (Giovagnoli et al., 2005, Lambon Ralph et al., 2010, Lambon Ralph et al., 2012). Further evidence comes from recent studies that have used fMRI to investigate remote effects of TMS to left ATL. These have found that TMS to left ATL reduces activation in this region but results in an increase in right ATL activation during semantic processing as well as significantly heightened inter-ATL functional connectivity (Binney and Lambon Ralph, 2015, Jung and Lambon Ralph, 2016). This suggests that increased activity in right ATL is able to compensate for impairment to left ATL, consistent with an integrated bilateral system. Computational investigations also indicate that a system in which the two ATLs co-operate to represent knowledge is advantageous because it is robust to unilateral damage (Schapiro et al., 2013).

Some researchers have assumed that conceptual representations in a bilateral ATL system are necessarily “amodal” and, as such, no functional specialisation is possible (Drane et al., 2013, Gainotti, 2012, Snowden et al., 2012). We do not share this view. Our proposition, supported by computational models, is that the content of representations in each ATL is influenced by (a) the inputs they receive from unimodal sensory and association cortices and (b) the outputs they provide to speech and motor production systems (Lambon Ralph et al., 2017, Plaut, 2002, Rice, Hoffman et al., 2015; Schapiro et al., 2013). This means that graded specialisations can develop within the bilateral ATL system as an emergent consequence of processing and connectivity asymmetries elsewhere in the brain. For the present study, two such asymmetries are relevant. First, speech production relies on a left-lateralised system of prefrontal and motor regions (Blank et al., 2002, Catani et al., 2007). Since intra-hemispheric connections are much more prevalent than inter-hemispheric ones, it follows that the left ATL is connected more strongly to speech output systems than the right ATL. This asymmetry in connectivity could cause the left ATL to play a greater role in the activation of speech output representation based on semantic information, an idea supported by implemented computational models (Lambon Ralph et al., 2001, Schapiro et al., 2013). Previous studies have found leftward naming biases in the lateral and superior ATL (Rice, Lambon Ralph et al, 2015). Here we have shown that this effect holds in the multimodal ventral portion of the ATL.

Another relevant hemispheric specialisation is the leftward bias for written word processing in posterior VTC (Cohen & Dehaene, 2004). This was present in our study, as was a right-hemisphere bias for pictured objects in the same area. This rightward bias for object processing has been found previously, but does not seem to be as prevalent as the well-known right-hemisphere bias for face processing (Nakamura et al., 2005, Seghier and Price, 2011, Thierry and Price, 2006). The left lateralisation we found for words in vATL is likely a downstream consequence of these asymmetries in higher-order visual cortex. Since left vATL receives inputs from left posterior VTC and right vATL does not, it appears to play a greater role in processing meaning from written stimuli. It is interesting to note that we did not observe right-hemisphere vATL specialisation for object stimuli, despite seeing effects in posterior VTC. Although this does not seem consistent with prior neuropsychological studies arguing for non-verbal specialisation in right ATL, it is important to note that many of these studies used faces as stimuli (e.g., Drane et al., 2013, Snowden et al., 2004, Snowden et al., 2012) and face processing may represent something of a special case. Right-biased activation in posterior VTC is especially pronounced for faces (Kanwisher & Yovel, 2006) and this strong bias may drive a stronger specialisation of function in ATL. Our data indicate a right-hemisphere bias is not present in vATL for other object categories, though direct comparisons between object and face recognition in this region would help to clarify the situation.

Our claim, then, is that left lateralisation for words in vATL is a downstream consequence of the lateralisation of visual processing in posterior VTC. The exact cause of this specialisation is debated but is likely to have its roots in the relationship between written word recognition and left-lateralised language production systems (Bouhali et al., 2014, Cai et al., 2010, Dundas et al., 2015, Price and Devlin, 2011). Indeed, Plaut and Behrmann (2011) presented a computational model of word and face recognition in which hemispheric specialisation in posterior VTC was an emergent consequence of this connectivity constraint. On their model, left and right VTC units received identical visual inputs but only the left VTC was connected to language output units. As a consequence, left VTC units developed graded specialisation for written word recognition and right VTC units subsequently came to specialise in face recognition. The basic principle here is similar to the one we set out earlier in respect to naming tasks, i.e., that left-hemisphere regions with the strongest connections to left-lateralised speech output systems tend to develop some specialisation for language-related processes. Specialisation in posterior VTC may be influenced by the strong quasi-regular mappings between orthography and speech sounds (Plaut, McClelland, Seidenberg, & Patterson, 1996), which appear to cause visual processing of written words to become biased towards the left hemisphere (Dehaene et al., 2010, Hashimoto and Sakai, 2004, Mei et al., 2013).

The present study has focused on the comprehension of verbal and non-verbal visual stimuli and not on auditory processing. In fact, few studies have investigated vATL activation for spoken word processing. The results of those that have are consistent with bilateral vATL activation, but with a graded left-hemisphere bias similar to that observed for written words (Halai et al., 2015, Spitsyna et al., 2006, Visser and Lambon Ralph, 2011). However, since laterality effects were not the main focus of any of these studies, direct statistical comparisons of left and right vATL activation were not performed, so no strong conclusions can be drawn. Based on connectivity-driven theory of ATL function, the degree to which biases are present will depend on the degree to which auditory processing for spoken words is lateralised to the left hemisphere (Hickok and Poeppel, 2007, Scott et al., 2000).

Finally, while in the present study we have been concerned with functional specialisation within the VTC specifically, it is worth noting that there are likely variations in function across the different parts of the ATL (e.g., ventral *vs* dorsolateral *vs* polar). In fact, the connectivity-based account of ATL function predicts graded functional specialisation across each ATL as a consequence of differential connectivity with sensory-motor and limbic cortices (Bajada et al., 2017, Binney et al., 2012, Rice, Hoffman et al., 2015). While the ATLs as a whole act as a multimodal conceptual hub, variations in the sensory-motor inputs received by different subregions of the ATLs can drive some specialisation in the type of information coded by each. For example, it appears that the dorsolateral ATL displays relative specialisation for auditory-verbal inputs, due to strong connectivity with posterior superior temporal cortex (Visser & Lambon Ralph, 2011) and the polar regions display relative specialisation for social and emotional concepts because they receive strong inputs from limbic areas (Binney et al., 2016, Ross and Olson, 2010). These within-ATL effects were beyond the scope of the present study. Nevertheless, a full understanding of hemispheric specialisation will need to take into account potential interactions between hemispheric effects and other graded effects across the region.
