# Supplementary material for: From percept to concept in the ventral temporal lobes: Graded hemispheric specialisation based on stimulus and task
Source: Cortex. 2018 Apr;101:107–18. doi: 10.1016/j.cortex.2018.01.015 (PMC5885984; doi:10.1016/j.cortex.2018.01.015)

Supplementary Figure 1: Example trials for non-semantic tasks


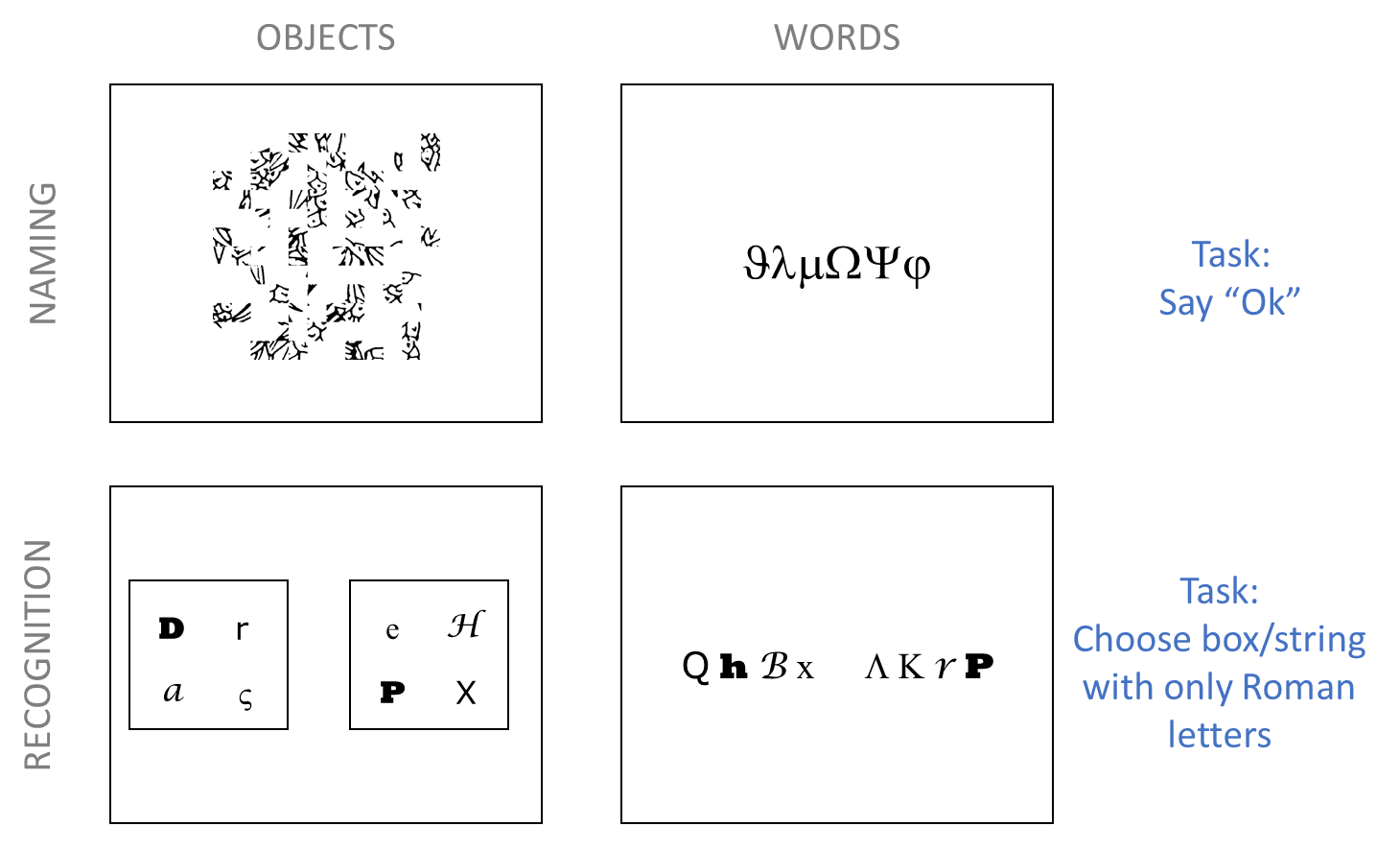


Supplementary Figure 2: TSNR averaged across all participants


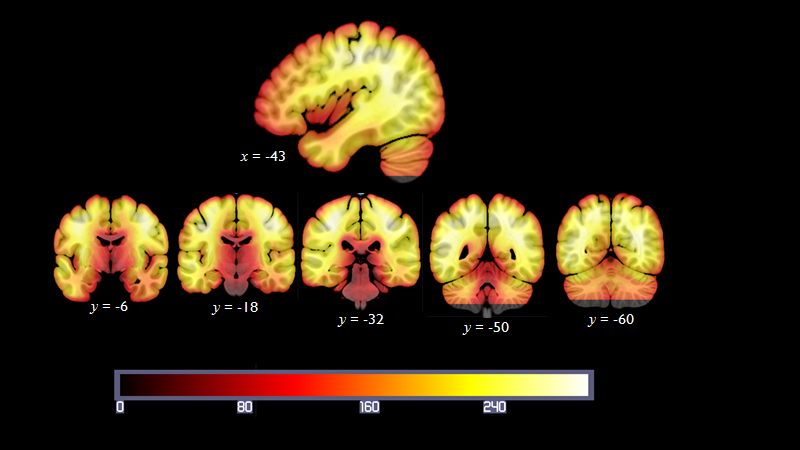

Supplement: Supplementary file 1 [file mmc1.docx]
